# Prussian Blue Analogs for Zinc Hybrid Ion Batteries: A Promising and Competitive Alternative to Aqueous Zinc‐Ion Batteries

**DOI:** 10.1002/EXP.20240180

**Published:** 2025-06-22

**Authors:** Shibo Meng, Dongshu Liu, Jinxiu Feng, Zhiyuan Zeng, Fangfang Wu, Wenxian Liu, Wenhui Shi, Xiehong Cao

**Affiliations:** ^1^ College of Materials Science and Engineering State Key Laboratory of Advanced Separation Membrane Materials and Pinghu Institute of Advanced Materials Zhejiang University of Technology Hangzhou China; ^2^ Department of Materials Science and Engineering and State Key Laboratory of Marine Pollution and Center of Super‐Diamond and Advanced Films City University of Hong Kong Hong Kong SAR China; ^3^ College of Chemical Engineering and State Key Laboratory of Advanced Separation Membrane Materials Zhejiang University of Technology Hangzhou China

**Keywords:** cathodes, hybrid ion batteries, metal‐organic frameworks, Prussian blue analogs, zinc batteries

## Abstract

Zinc hybrid ion batteries (ZHIBs) represent an innovative alternative building upon the strengths of zinc‐ion batteries (ZIBs) while overcoming their inherent challenges. ZHIBs employ a versatile strategy of utilizing monovalent ions as charge carriers, which not only accelerates the diffusion kinetics but also fortifies the structural integrity of the cathode materials. This approach effectively addresses the issues of slow divalent zinc ion migration and the strong coulombic interactions that have been noted in ZIBs. Prussian blue analogs (PBAs) are notable for their open framework, structural robustness, and adaptability to various ions. Despite their advantages, challenges such as the irreversible phase transitions upon cycling, low electrical conductivity, and abundant defects and crystal water impede the broader application of PBAs in ZHIBs. This review discusses the advantages of PBA cathodes, comprehensively summarizing the challenges encountered and proposing corresponding strategies aimed to these challenges. Recent applications of PBAs in ZHIBs are summarized, highlighting their potential and limitations. Finally, the review outlines the opportunities and challenges facing the field, proposing potential research pathways to further develop PBA cathodes for ZHIBs.

## Graphical abstract

Zinc hybrid ion batteries (ZHIBs) represent an innovative alternative to traditional zinc‐ion batteries. This review explores the benefits and hurdles specific to Prussian blue analog (PBA) cathodes in ZHIBs, along with current applications and their potential. Additionally, it highlights the field's opportunities and challenges, suggesting paths for future research to enhance PBA cathode development in ZHIBs.

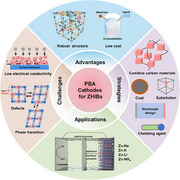

## 1 Introduction

With the increasingly severe global environmental problems, the importance of adopting cleaner energy sources such as wind, solar, and hydrogen has been recognized [1]. To foster a greener world, it is essential to leverage these clean energy sources [2]. Nonetheless, the intermittency of these energy sources necessitates the development of advanced energy storage and transport systems [3]. Lithium‐ion batteries (LIBs) have dominated the market for decades due to their high energy density (200–250 Wh kg^−1^) and satisfactory cycle life [4]. However, the high cost of lithium and associated safety issues limit their further application [5]. It is crucial to develop novel battery technology with safer systems and cost‐effective metal anodes. Aqueous batteries, which employ aqueous solution as their electrolyte, exhibit high levels of safety and ionic conductivity when compared to battery systems utilizing organic solvents, such as lithium‐, sodium‐, and potassium‐ion batteries [6]. Consequently, aqueous batteries have garnered significant attention as potential candidates for large‐scale energy storage applications. Among various rechargeable aqueous metal‐ion batteries, zinc‐ion batteries (ZIBs) have particularly emerged as promising alternatives for grid‐scale electrochemical energy storage systems [7]. ZIBs are favored for their high theoretical specific capacity, low redox potential, and cost‐effective zinc metal, making them a research hotspot in recent years [8].

**FIGURE 1:**
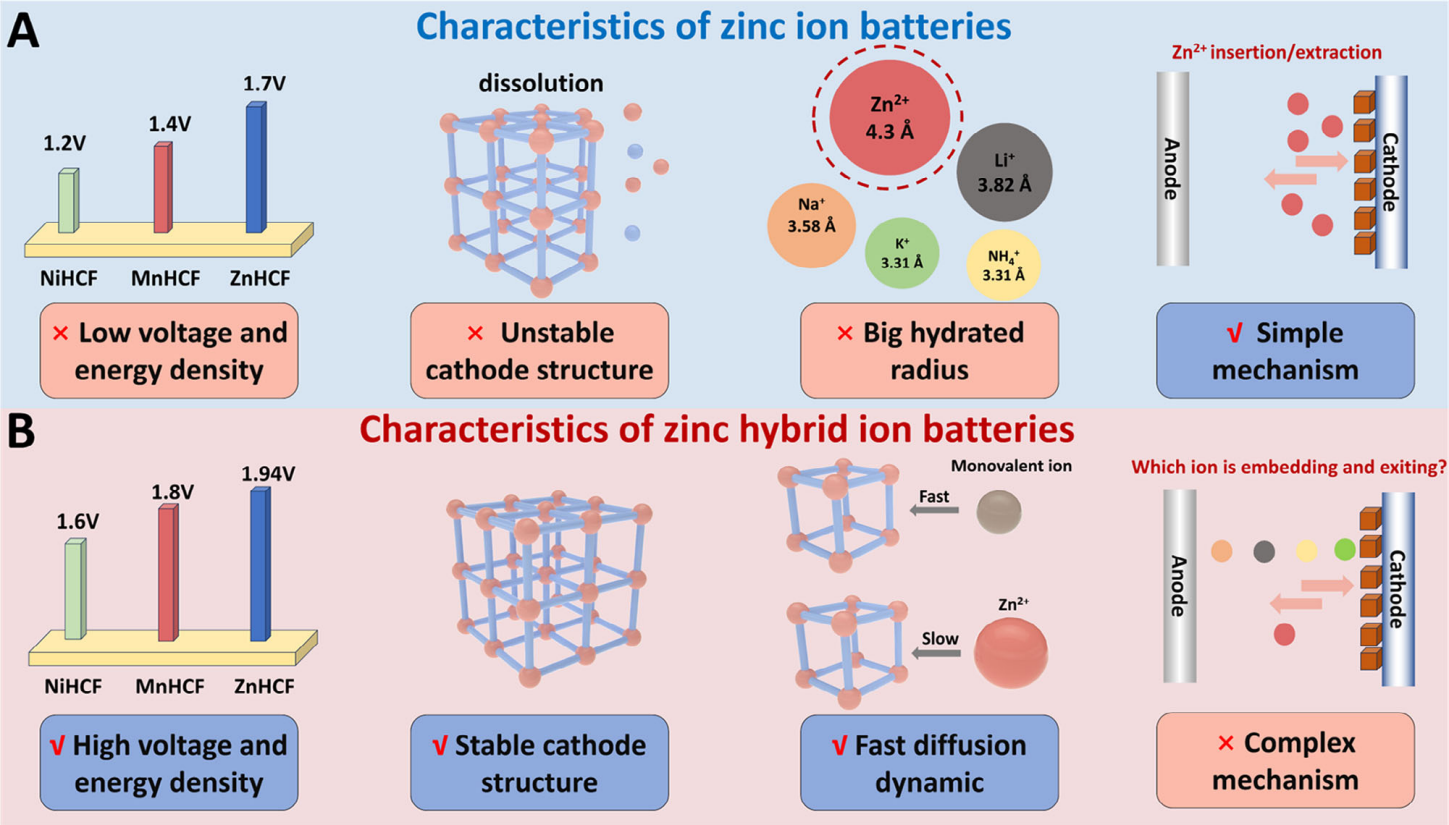
Schematic diagrams illustrating the characteristics of (A) zinc ion batteries using PBAs as cathode. (B) zinc hybrid ion batteries using PBAs as cathode.

PBAs stand out as the most promising cathodes among various cathode materials for ZIBs, due to their open framework and low cost. Despite considerable progress, ZIBs using PBAs as cathodes still face challenges such as relatively low voltage and energy density, which impede their commercialization (Figure 1A). This is attributed to the large Stokes radius of Zn^2+^ ions and their strong coulombic interactions with cathode materials, leading to sluggish intercalation kinetics and poor structure stability [9]. In contrast, ZHIBs offer enhanced performance over ZIBs in terms of operating voltage, energy density, cathode stability, and ion diffusion dynamics (Figure 1B). This may owe to the smaller Stokes radius and weaker electrostatic interaction of monovalent ions, presenting a promising avenue for advancing battery technology.

**FIGURE 2:**
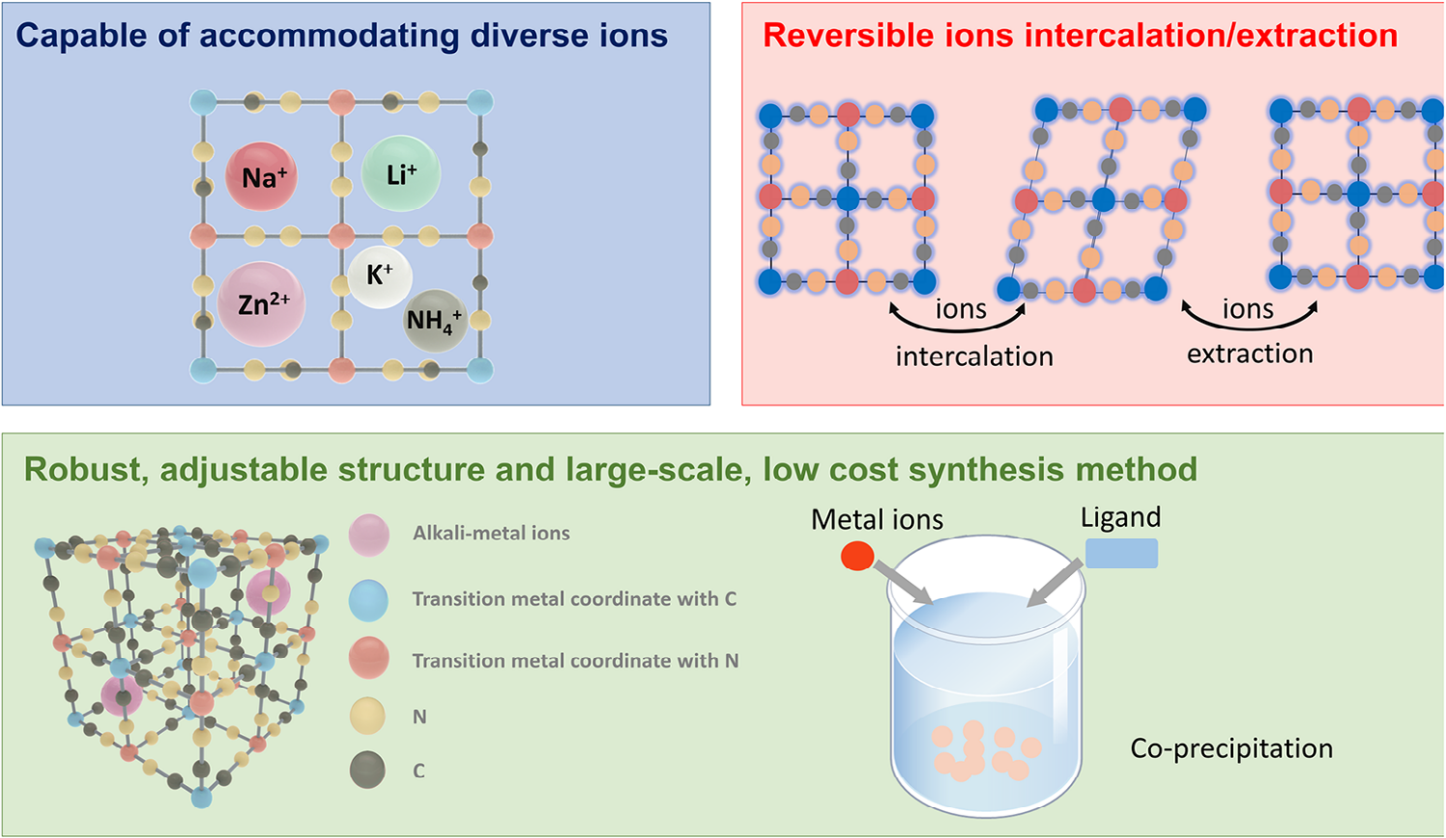
The advantages of PBA cathodes.

The ZHIBs system, assembling with a LiMn_2_O_4_ cathode, an aqueous electrolyte comprising ZnCl_2_ and LiCl, and a zinc metal anode, was first reported by Yan et al. in 2012 [10]. This pioneering system employed a dual‐ion strategy, where Li^+^ ions facilitated intercalation/extraction processes at the cathode, while Zn^2+^ ions underwent deposition/dissolution at the anode. This work has inspired subsequent research into diversified ZHIB configurations, including zinc‐sodium hybrid ion batteries (ZSHIBs), zinc‐potassium hybrid ion batteries (ZPHIBs), zinc‐lithium hybrid ion batteries (ZLHIBs), and zinc‐ammonium hybrid ion batteries (ZAHIBs). The versatility of ZHIBs has allowed for the integration of cathodes typically reserved for monovalent ion batteries into zinc‐based systems, extending to materials like LiMn_2_O_4_ [11], LiFePO_4_ [12], Na_3_V_2_(PO_4_)_3_ [13], graphite [14] and Prussian blue analogs (PBAs) [15], etc. Among these cathode materials, PBAs have received considerable attention due to their open framework structure, high diffusion coefficient (10^−9^ to 10^−8^ cm^2^ s^−1^), and ability to host multiple ions, including Li^+^, Na^+^, K^+^, NH_4_^+^, and Zn^2+^ [16]. Meanwhile, their structural integrity remains largely unchanged during ion insertion, which ensures a long cycle life for the batteries. The structural formula of PBAs, generally denoted as A*_X_* M_A_[M_B_(CN)_6_]_1−z_γ*_z_* ·*n*H_2_O (0 < *x* < 2; 0< *z* < 1), reflects their compositional adaptability and tunable electrochemical properties [17]. The straightforward energy storage mechanism of PBAs, based on reversible ion intercalation/extraction, supports efficient operation across various ZHIB configurations. Moreover, the synthesis of PBAs via room temperature co‐precipitation, coupled with the economic accessibility of raw materials, facilitates scalable production processes (Figure 2). These advantages make PBAs a promising choice for cathode materials in versatile battery systems. Recently, PBA cathodes have emerged as the mainstream cathode material for sodium/potassium‐ion batteries (SIBs/PIBs) due to their advantages of high energy density and low cost. However, the persistent crystalline water within PBA cathodes poses a major obstacle to their further development in SIBs/PIBs [18]. Consequently, numerous scientists have dedicated efforts to reducing the amount of crystalline water in PBAs, proposing various methods, including two major categories, namely, regulating the crystallization process and subsequent defect repair [19]. With the advancement of ZHIBs, an increasing number of studies have proved that PBAs are also competitive cathode candidates for ZHIBs and have underscored their viability for large‐scale energy storage applications [20].

Despite previous reviews that have discussed PBA cathodes in zinc‐based batteries [21], focusing on the synthesis and modification of cathodes, the literature has largely bypassed the pivotal role of PBAs in ZHIBs. Given that the efficacy of the batteries hinges critically on the nature of the charge carriers, a systematic review summarizing the recent strides in advancements of PBA cathodes for ZHIBs is not only timely but also essential. This review aims to provide a comprehensive overview of PBA cathodes utilized in various ZHIB configurations, offering critical insights into the challenges and potential research avenues for future research.

## 2 Zinc Hybrid Ion Batteries

**FIGURE 3:**
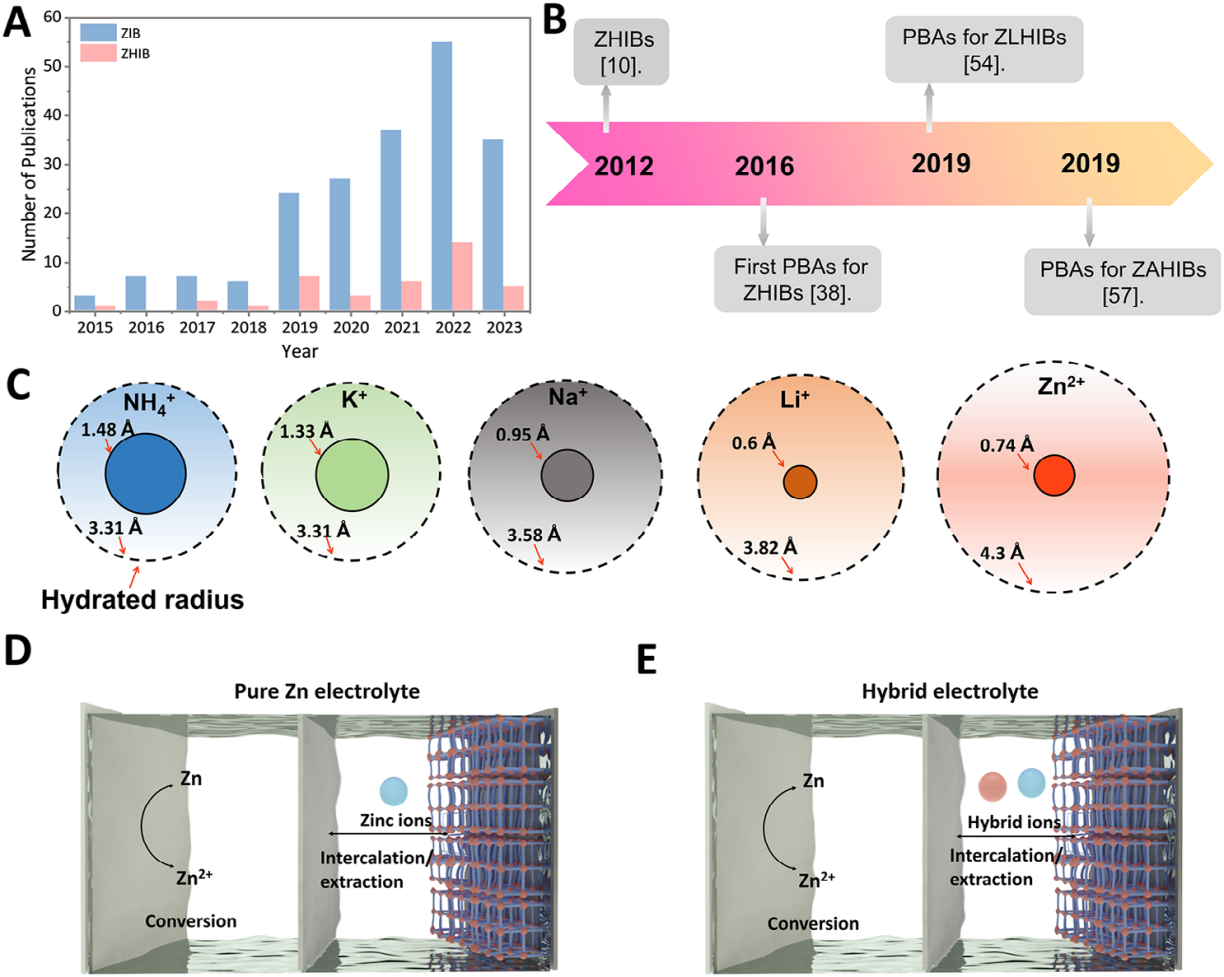
(A) Recent trend of publications focusing on ZIBs and ZHIBs. Data collected from the Clarivate Web of Science. (B) The emerged new type of ZHIBs. (C) The comparison of ionic radius and hydrated radius of NH_4_^+^, K^+^, Na^+^, Li^+^, and Zn^2+^. Schematic diagrams illustrating the configuration and mechanism of (D) ZIBs and (E) ZHIBs.

Current research on PBA cathodes in zinc‐based energy storage predominantly focuses on ZIBs [22]. ZHIBs have recently come to the forefront, offering notable improvements in electrochemical performance (Figure 3A). A multitude of researchers have recognized the great potential of ZHIBs, contributing to and accelerating the breakthrough in this area (Figure 3B). Generally, the energy storage mechanism of the PBA cathodes relies on the reversible insertion and extraction of ions, with their electrochemical behavior significantly influenced by the types and properties of charge carriers. In aqueous electrolyte, monovalent ions usually have a smaller hydrated radius, facilitating the rapid diffusion of batteries (Figure 3C). ZHIBs introduce additional monovalent ions into the electrolyte, which facilitates the insertion and extraction processes at the PBA cathodes without affecting the operation of the zinc anode (Figure 3D,E). This hybrid system can not only enhance the working voltage of the PBA cathodes but also maximize the utilization of the PBA cathodes lattice space, offering advantages over traditional ZIB systems. While the deployment of PBA cathodes in ZHIBs represents a specialized area of study, it is essential to summarize the past progress while also delineating future research directions to advance this promising technology.

## 3 Challenges and Strategies for PBA Cathodes

**FIGURE 4:**
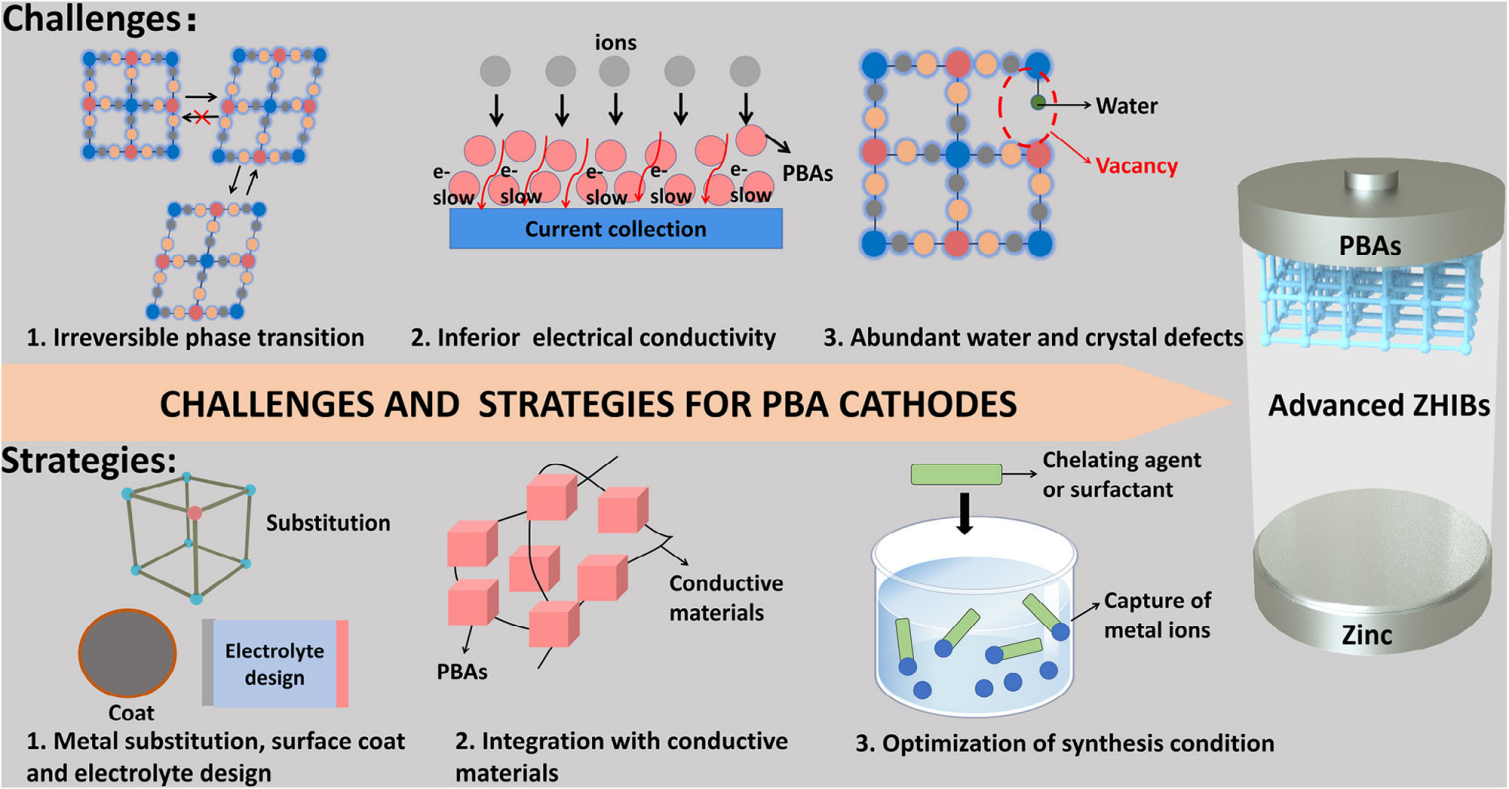
The challenges and strategies for PBA cathodes.

The number of redox active centers possessed by PBAs directly affects the specific capacity and energy density of batteries. Hence, most research has concentrated on PBA cathodes with dual active sites. While PBAs with more active centers can theoretically achieve higher capacities, they also experience more pronounced lattice contraction and expansion during charge–discharge processes [23]. These mechanical stresses can lead to irreversible phase transitions, severely compromising cycling stability and voltage retention. Additionally, the presence of Fe[CN]_6_ vacancies and crystal water within the structure of PBAs can further accelerate the cathode dissolution and degrade cycling stability [24, 29]. Another challenge is the inherent poor conductivity of PBAs, which impairs the rate of migration of ions and electrons, thus limiting their rate performance [25]. Recently, various modification strategies have been proposed to address these issues of PBA cathodes. In this section, these strategies are summarized and divided into three categories (Figure 4), which are inhibition of irreversible phase transitions, integration with conductive materials, and optimization of synthesis conditions.

### 3.1 Inhibition of Irreversible Phase Transition

The cycling stability of PBA cathodes is closely related to their crystal composition and structure. Transition metal (TM) substitution is one of the most common and effective methods to suppress the irreversible phase transitions of PBA cathodes. TM substitution typically involves the partial replacement of active metal elements (such as Mn, Fe, and Co) by one or more electrochemically inert metal elements including Ni, Cu, Zn, etc. This approach can effectively alleviate lattice expansion during ion insertion/extraction and stabilize the lattice structure of PBAs. Surface modification is another widely used method to suppress phase transitions and improve stability. Through appropriate modification of PBAs materials, artificial electrode electrolyte interface (EEI) layers can be formed on their surfaces, which greatly improve the stability of PBA cathodes and their wettability with electrolytes. In addition, electrolyte modification is also an effective method for stabilizing PBAs against phase transitions.

As a typical example, Lou and coworkers prepared copper‐substituted manganese hexacyanoferrate (Mn‐PBA) double shelled nanoboxes (CuMn‐PBA DSNBs) by tannic acid etching and cation exchange methods and investigated the stabilizing effects of partial copper substitution [26]. Their findings indicated that the copper substitution can not only effectively suppress the Jahn–Teller distortions but also improve the cycling and rate performance of the cathodes. In addition, the tannic acid etching induced the formation of manganese vacancies, which contributed to phase transition suppression, lattice strain reduction, and an increase in active sites. As a result, the CuMn‐PBA DSNBs exhibited a good cycling stability with a capacity retention of 96.8% after 2000 cycles at 1 A g^−1^ [27].

Regarding to surface coating, Wu et al. recently demonstrated that PBA cathodes could achieve stable cycling in alkaline environments by applying a nickel/carbon (Ni/C) nanoparticle‐based coating on the Na_2_MnFe(CN)_6_ (NMF) cathode [28]. This coating effectively eliminates the need for expensive additives like fluorine salt, “water‐in‐salt” (WIS) electrolytes, and organic co‐solvents, while maintaining the structural integrity of PBAs. In situ attenuated total reflectance‐infrared (ATR‐IR) spectroscopy revealed that at potentials above 0.6 V, the modified NMF cathode exhibited new peaks at 1798 and 2023 cm^−1^, attributed to two asymmetric O─H stretching modes of H_3_O^+^. This indicates a Ni/C‐induced rich H_3_O^+^ interface, which stabilizes the NMF structure in alkaline conditions. Operando differential electrochemical mass spectrometry (DEMS) analysis confirmed that this coating allows the NMF cathode to operate reliably in alkaline media without noticeable hydrogen or oxygen evolution reactions.

Electrolyte design is also critical for inhibiting irreversible phase transitions in PBA cathodes. For example, Li and coworkers developed a new electrolyte featuring a weakly solvating effect (WSE), by incorporation DX (1,4‐Dioxane) with a low polarity and DN (donor number) as a co‐solvent, which forms an anion‐rich solvation structure. The Raman spectra of ‐SO_3_H stretching bands confirmed the WSE, resulting in the presence of OTF^−^ anions predominantly in the CIP/AGG configuration (about 52.2% fraction). Using this electrolyte, the FeMn‐PBA||Zn battery displayed excellent rate performance, maintaining 62.3% of its initial capacity even when the current density was increased to 8 A g^−1^ [15]. Meanwhile, the full FeMn‐PBA||Zn battery exhibited high charge/discharge plateaus of 2.1 and 1.9 V, alongside remarkable cycling stability, retaining 99.3% of its initial capacity after 5000 cycles with the Zn/Na WSE electrolyte. Ji et al. prepared a double‐network hydrogel electrolyte by a synergy of H‐bonding and polymerization to achieve a high voltage ZnHCF||Zn hybrid cell [29]. Due to the abundant hydrophilic groups, such as carboxyl groups, present in the network of hydrogel, water activity is effectively restrained, thereby enabling the achievement of remarkable performance characterized by a discharge voltage as high as 1.8 V and a stable cycling performance for up to 800 cycles.

### 3.2 Integration With Conductive Materials

The electrical conductivity of PBA cathodes critically influences battery performance. Effective charge and discharge cycles require the transfer of carriers from the PBAs to the external circuit, necessitating high electrical conductivity within the PBAs. A prevalent method to boost the electrical conductivity involves forming composites with conductive materials, such as carbon‐based materials (e.g., graphene, carbon nanotubes, and carbon dots) and conductive polymers (e.g., polypyrrole and polyaniline). For example, You and coworkers prepared a composite wherein PB nanocrystals are interlinked by carbon nanotubes (PB/CNT). By employing a CNT network as the nucleation site, the PB nanocubes are monodispersed within the CNT network, with individual CNTs traversing through the PB to create a necklace‐like composite structure. The synergy between the PB nanocrystals and CNTs facilitates rapid ionic and electronic transport kinetics, enabling the composite cathode to deliver a high capacity of 142 mAh g^−1^ and an impressive specific energy density of 408 Wh kg^−1^ [30]. In addition, with the integration of conductive polymers is another effective strategy for enhancing electrical conductivity. For example, Chen et al. synthesized a composite of potassium manganese hexacyanoferrate with polypyrrole (KMHCF@PPY) using an in situ polymerization technique. Benefiting from the intimately bonded PPy conductive coating, KMHCF@PPY possesses a better rate performance of 57.5 mAh g^−1^ at 500 mA g^−1^. Additionally, this composite material demonstrates a high discharge capacity of 107.6 mAh g^−1^ at a current density of 100 mA g^−1^ [31]. These examples highlight the significant advancements in improving the electrical performance of PBA cathodes through innovative composite approaches.

### 3.3 Optimization of Synthesis Condition

PBAs are typically synthesized via a co‐precipitation method in aqueous solutions, where metal ions and ligands rapidly coordinate to form low‐solubility metal‐organic framework materials [32]. However, this rapid precipitation often leads to the formation of crystal defects and the inclusion of crystallization water. The presence of Fe(CN)_6_ vacancies (V_FeCN_) and water in the crystal structure is known to degrade cycling performance and limit structural reversibility [33]. Based on previous research, to synthesize high‐quality PBA crystallites, approaches such as the use of chelating agents or surfactants, adjustment of aging temperatures have been developed.

One effective approach involves the addition of chelating agents or surfactants to the precursor solution, which slows down the crystallization rate to obtain the high crystallinity PBAs. For instance, Deng et al. utilized ethylenediaminetetraacetic acid dipotassium salt (EDTA‐2K) as a chelating agent in the synthesis of potassium manganese hexacyanoferrate (K_2_Mn[Fe(CN)_6_]) [34]. This method reduced the V_FeCN_ content to 8% relative to Mn, and slowed down the nucleation and growth of crystals, resulting in well‐crystallized PBAs with low water content. The perfect crystal structure was further supported by theoretical calculations and inductively coupled plasma‐mass spectrometry (ICP‐MS), confirming the detrimental role of defects and crystallization water in metal ion dissolution and decreased cycling performance. Similarly, Zhan et al. employed EDTA‐2K as a chelating agent and obtained a high crystallinity and low water content in KMnHCF, which exhibited low Mn dissolution and a stable cathode electrolyte interface (CEI) [35]. When used in ZPHIBs with an electrolyte mixture of 0.5 M KCF_3_SO_3_ and 0.1 M Zn(CF_3_SO_3_)_2_, the battery demonstrated a high capacity and an exceptionally long cycle life of 15,000 cycles.

Adjusting the aging temperature is another way to reduce V_FeCN_ content, as temperature influences the crystallization kinetics and defect formation. Wang and coworkers reported a thermal repair method that effectively reduced V_FeCN_ content to 2%, localized within the interior of the PBAs, while also removing water at 90°C [36]. This process involved the gradual reduction [Fe^III^(CN)_6_] in the lattice of precursors to [Fe^II^(CN)_6_] at elevated temperatures and the subsequent substitution of Fe^II^(CN)_6_^4−^ for surface V_FeCN_. The concentrated distribution of V_FeCN_ significantly inhibited surface Mn dissolution and contributed to a dense cathode electrolyte interface (CEI). Consequently, the KMnHCF exhibited a high initial discharge capacity of 108 mAh g^−1^ and maintained a capacity retention of 95% over 2000 cycles. Additionally, other innovative methods have been employed to achieve well‐crystallized PBAs. For instance, Li et al. fabricated spindle‐like PW (K_1.72_Fe[Fe(CN)_6_]_0.96_·0.342H_2_O) via a template‐engaged reduction method [37]. The resulting hierarchical porous structure, inherited from the metal‐organic frameworks, provides abundant active sites and facilitates fast ion diffusion kinetics.

## 4 Zinc‐Sodium Hybrid Ion Batteries

Possessing excellent compatibility between Na ions and the pores of PBAs, PBA cathodes have been widely studied in both aqueous and nonaqueous sodium‐ion batteries. Their versatility also extends to ZHIBs, which benefit significantly from the attributes of PBA cathodes.

**FIGURE 5:**
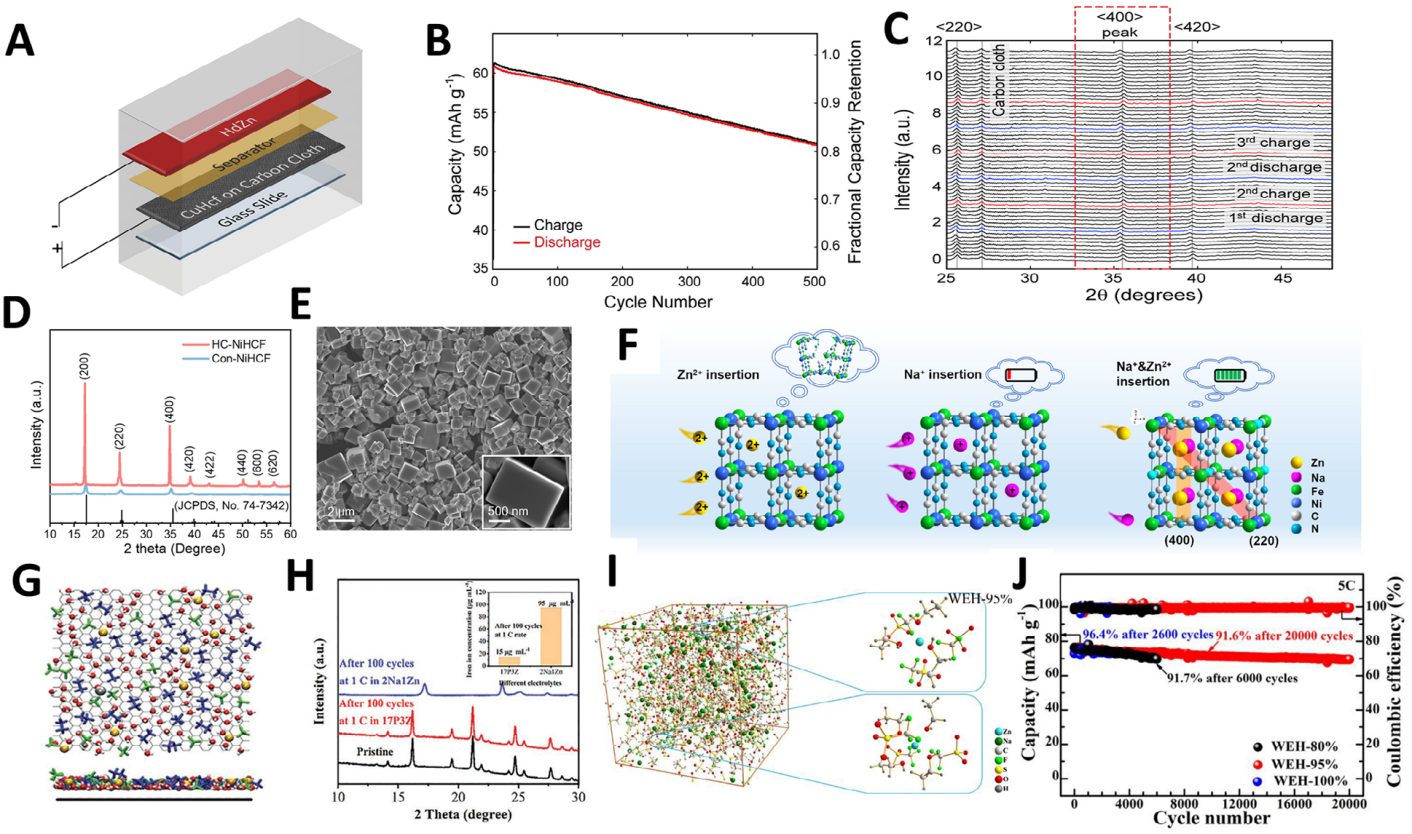
(A) Schematic figure of ZSHIBs. (B) Cycling performance of ZSHIBs. (C) In situ XRD patterns observed during three cycles at 3C rate. Reproduced with permission [38]. Copyright 2015, Elsevier. (D) XRD comparison between hydrothermally crystallized NiHCF (HC‐NiHCF) and conventionally synthesized NiHCF (Con‐NiHCF). (E) SEM images of HC‐NiHCF. (F) Schematic illustration of Na^+^ ions and Zn^2+^ ion insertion into HC‐NiHCF. Reproduced with permission [40]. Copyright 2023, Elsevier. (G) Molecular dynamics snapshots showing the double layer structure of the 17 P 3 Z electrolyte at the graphite interface. (H) XRD patterns of the ZnHCF electrode before and after 100 cycles. Reproduced with permission [43]. Copyright 2021, Wiley. (I) Snapshot boxes and Zn^2+^ solvation sheath structure of the WEH‐95% electrolyte. (J) Cycling performance comparison at a 5C rate using WEH‐80%, WEH‐95%, and WEH‐100% electrolytes. Reproduced with permission [44]. Copyright 2022, Elsevier.

In 2016, Steingart and coworkers first identified that the degradation in cycle life of PBA cathodes was due to the irreversible intercalation and extraction of divalent zinc ions, which damages the crystal structure of the cathode [38]. To mitigate this issue, a strategy was developed that involved the insertion and extraction of sodium ions within the PBA lattice, a process less disruptive than that involving zinc ions. By utilizing hyper‐dendritic zinc as an anode, copper hexacyanoferrate (CuHCF) as a cathode, and a 1 M Na_2_SO_4_ + 0.01 M H_2_SO_4_ electrolyte, the first ZSHIB was assembled with a CuHCF cathode (Figure 5A). This prototype exhibited an impressive capacity retention of 83% after 500 cycles at a discharge rate of 5C (Figure 5B). Notably, this setup was home‐assembled and not sealed in a coin‐cell, which helped minimize gas production during charging to 2.1 V. In situ X‐ray diffraction investigations revealed that Na^+^ ions preferentially intercalated within CuHCF's open crystal structure, thereby reducing Zn^2+^ ion intercalation (Figure 5C).

In sodium‐ion batteries, rapid reaction rates of conventional co‐precipitation methods lead to defects, such as [Fe(CN)_6_] vacancies, sodium deficiency, and low crystallinity, leading to poor electrochemical performance [39]. In aqueous systems, low crystallinity further exacerbates structure collapse during cycling. To address these problems, Wang et al. prepared high crystallinity cubic shaped nickel hexacyanoferrate (HC‐NiHCF) using ethylenediaminetetraacetic acid (EDTA) as a chelating agent and polyvinylpyrrolidone (PVP) as a surfactant (Figure 5D) [40]. HC‐NiHCF with high crystallinity exhibits a well‐defined square morphology due to its slow nucleation and growth process (Figure 5E). When assembled with a mixed electrolyte of 1 M Na_2_SO_4_ + 1 M ZnSO_4_, the full battery exhibited superior electrochemical performances, delivering a high specific capacity of 73.9 mAh g^−1^ at 0.1 A g^−1^ and excellent cycling stability with 75% capacity retention over 1000 cycles at 2 A g^−1^. This study also proposed a co‐intercalation mechanism for the PBA cathodes in ZSHIBs (Figure 5F), where the co‐insertion of Zn^2+^ and Na^+^ ions into the NiHCF lattice resulted in higher capacity and improved cycling performance.

Another common issue with PBA cathodes in ZSHIBs is severe capacity deterioration in aqueous electrolyte. The PBA structure, which relies on the coordination between transition metals and nitrogen atoms in Fe‐CN groups, is susceptible to repeated lattice stress and water‐induced dissolution [41]. Highly polar water molecules can aggressively attack the cathode material, causing rapid capacity fade of the battery [42]. A promising solution to mitigate these effects is the utilization of highly concentrated electrolytes. Qian and coworkers reported that a concentrated aqueous electrolyte (17 M NaClO_4_ + 3 M ZnOTf) significantly suppressed the degradation of the zinc hexacyanoferrate (ZnHCF) cathode. With this electrolyte, the ZnHCF||Zn battery exhibited a remarkable 98% capacity retention after 2000 cycles at 5C rates [43]. The high anion concentration at the Helmholtz plane effectively shields the cathode from the aqueous environment, thus slowing down PBA degradation (Figure 5G,H).

Despite their effectiveness, highly concentrated WIS electrolytes often suffer from high cost and viscosity, which possess low ionic conductivity and hinders their practical application. An alternative approach to address water‐related degradation involves incorporating additional components, typically organic solvents, into the aqueous electrolyte. For example, Sun et al. proposed an economical water/ethanol hybrid (1 M NaOTf + 0.1 M Zn(OTf)_2_ in 95 wt% ethanol) electrolyte that enabled remarkably stable cycling, retaining 91.6% capacity after 20,000 cycles at a 5C rate (Figure 5I,J) [44]. However, it is crucial to maintain the organic solvent content within a safe threshold to ensure the integrity of the electrolyte system.

## 5 Zinc‐Potassium Hybrid Ion Batteries

Among various battery systems, potassium‐ion batteries (PIB) have received significant attention due to the advantages standard redox potential of potassium ions (−2.93 V vs. SHE) and the relative abundance of potassium compared to lithium. However, PIBs face challenges of the large ionic radius of K^+^ ion (1.33 Å), which is substantially larger than that of Li^+^ (0.6 Å) and Na^+^ (0.95 Å), potentially complicating the insertion and extraction processes. Despite this, the Stokes radius of hydrated potassium (3.31 Å) is smaller than that of Li^+^ (3.82 Å) and Na^+^ (3.58 Å), thereby resulting in a stronger ion conductivity in hydrated potassium‐ion batteries (APIBs). Nevertheless, the high reactivity of potassium metal with aqueous electrolytes prevents its use as an anode in APIBs. Therefore, ZPHIBs using zinc anodes and PBA cathodes represent a viable alternative.

**FIGURE 6:**
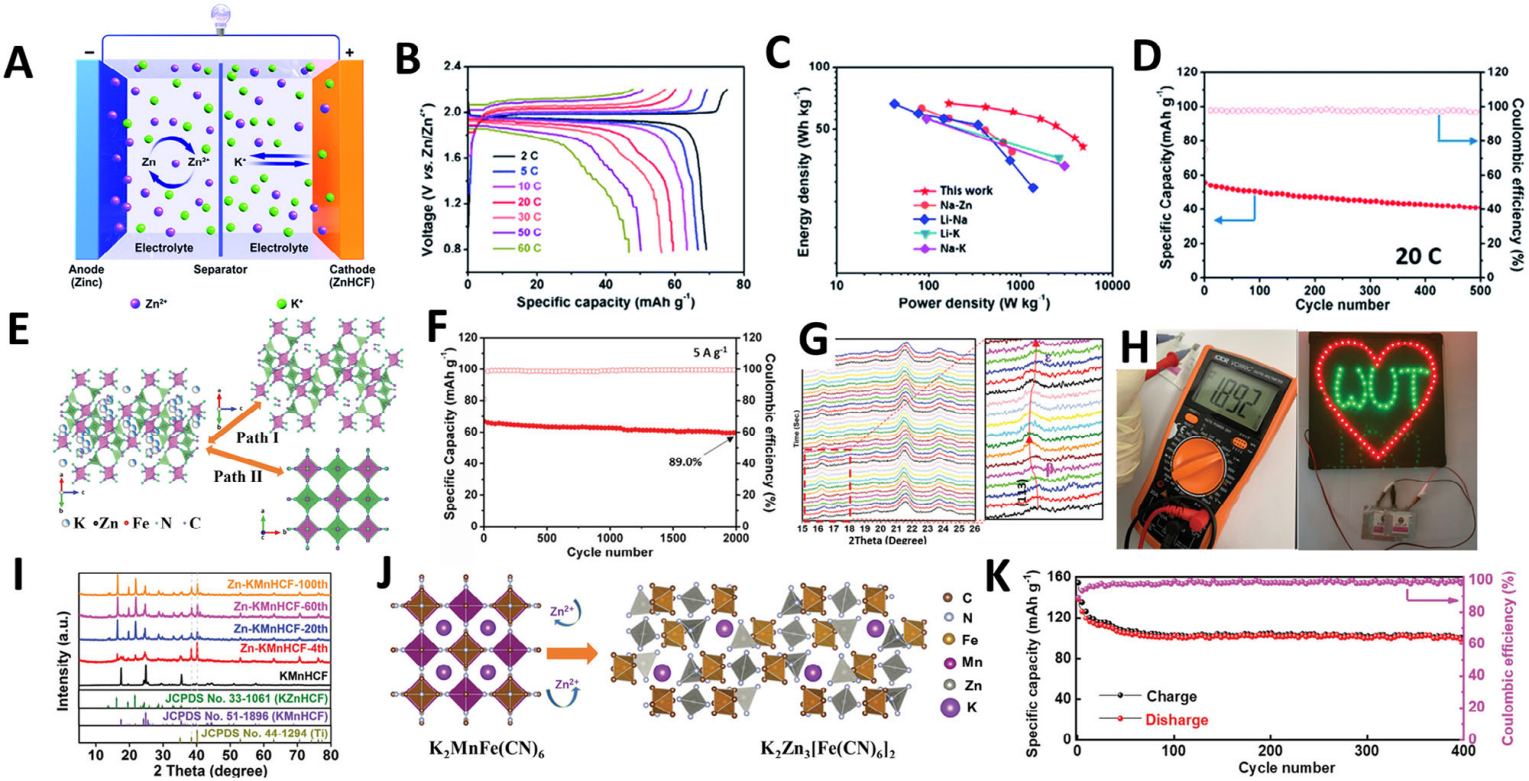
(A) Schematic diagram of ZPHIBs. (B) GCD profiles of the ZnHCF||Zn cell. (C) Ragone plot of the ZnHCF||Zn cell. (D) Cycling performance of the ZnHCF||Zn cell. Reproduced with permission [45]. Copyright 2022, Elsevier. (E) Electrolyte dependent mechanisms in ZPHIBs: Path I (solid solution phase, happens in high concentration of K^+^ and K^+^/Zn^2+^ hybrid electrolytes) and path II (two‐phase transition in low concentration of K^+^ electrolyte). (F) Cycling performance of the KZnHCF||Zn coin cell. (G) In situ XRD patterns of the cathode during cycling. (H) The open circuit voltage of the pouch cell and the lighted LED screen by two pouch cells. Reproduced with permission [46]. Copyright 2021, Wiley. (I) The XRD patterns of KMnHCF electrode for KMnHCF||Zn batteries over cycling. (J) Schematic figure of the phase transformation for KMnHCF electrode. (K) Cycling performance of the KMnHCF||Zn battery at 0.2 A g^−1^. Reproduced with permission [48]. Copyright 2021, Wiley.

Mai and coworkers pioneered the use of zinc hexacyanoferrate (ZnHCF) as cathode materials for ZPHIBs, achieving notable performance with a high discharge voltage of 1.937 V and an impressive power density of 4.76 kW kg^−1^ (Figure 6A–D) [45]. Their study utilized in situ XRD to monitor phase transitions of ZnHCF in different electrolytes, revealing that the higher charge density of Zn^2+^ ions was more likely to initiate a structural transformation from rhombohedral to cubic. In contrast, the insertion/extraction of K^+^ ions exhibited a solid‐solution mechanism. By varying the electrolyte concentrations, Mai and coworkers identified that certain concentrations lead to detrimental phase transitions, reducing cycle life, while others maintain crystallographic stability, improving cyclability, and kinetics. By optimizing the electrolyte concentration in a mixed solution of 5.0 m KCF_3_SO_3_ and 3.0 m KCF_3_SO_3_ with 2.0 m Zn(CF_3_SO_3_)_2_, they achieved remarkable long‐term cycling performance, surpassing 2000 cycles (Figure 6E–G) [46]. In addition, they assembled a pouch cell that exhibited a high open‐circuit voltage of 1.892 V, successfully powering 102 LED bulbs with two pouch cells connected in series (Figure 6H).

Despite the stable cycling performance of ZnHCF, its limitation to one electron reaction constrains further capacity increase. To enhance the energy density of ZPHIBs, exploration of other Prussian blue analogs capable of facilitating double electron transfer, such as Fe‐PBAs, Mn‐PBAs, and Co‐PBAs (M_1_ = Fe, Mn, Co, M_2_ = Fe) is necessary [47]. Deng et al. reported a high‐capacity potassium manganese hexacyanoferrate cathode (K_1.6_Mn[Fe(CN)_6_]_0.94_·0.63H_2_O) for ZPHIBs, but noted instability in MnHCF during cycling, which gradually transitioned into ZnHCF (Figure 6I) [48]. Density functional theory (DFT) calculations and density of states (DOS) analyses suggested that this phase change was driven by the Jahn–Teller effect of Mn^4+^ exacerbated by Zn^2+^ incorporation, resulting in expanded ionic channels within the ZnHCF structure (Figure 6J). The KMnHCF||Zn cell initially demonstrated a capacity of 138 mAh g^−1^, which stabilized at around 100 mAh g^−1^ over 400 cycles at a current density of 0.2 A g^−1^ (Figure 6K). Despite using highly concentrated WIS electrolytes to inhibit phase transitions, the gradual transition from MnHCF to ZnHCF persisted.

**FIGURE 7:**
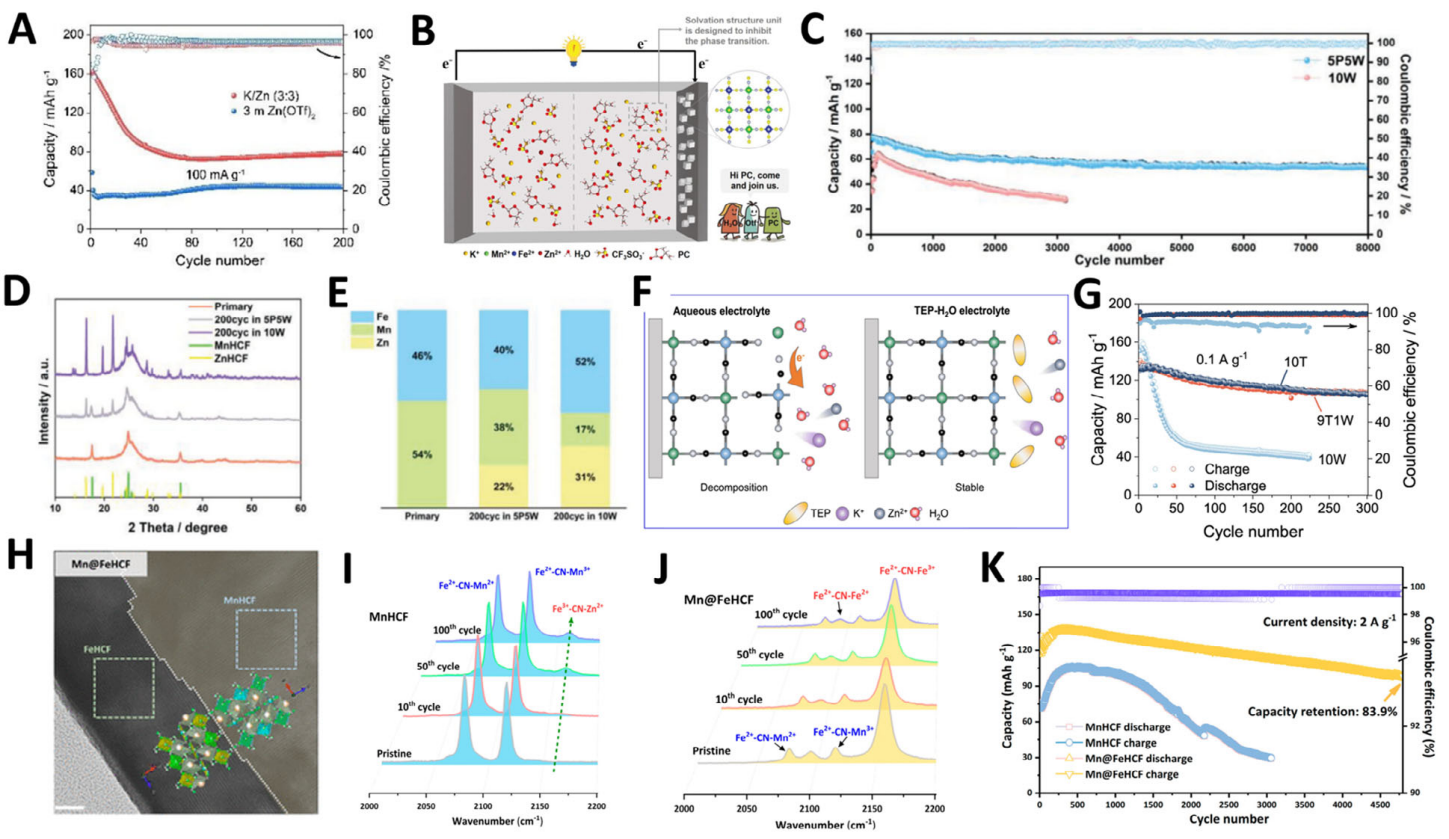
(A) Cycling performance of FeHCF in two different electrolytes. Reproduced with permission [49]. Copyright 2021, Wiley. (B) Schematic representation of MnHCF||Zn hybrid battery with a PC‐H_2_O co‐solvent containing K^+^/Zn^2+^ hybrid electrolyte. (C) Cycling performance of MnHCF||Zn hybrid batteries with 5 P 5 W （50vol% PC/water） and 10 W （100vol% water） electrolytes at 2.5 A g^−1^. (D) XRD patterns of MnHCF electrode at pristine and after 200 cycles in 5 P 5 W and 10 W electrolytes. (E) ICP analysis of MnHCF electrode at pristine and after 200 cycles in 5 P 5 W and 10 W electrolytes. Reproduced with permission [50]. Copyright 2023, Wiley. (F) Schematic figure of the surface reduction on the fully charged FeHCF cathode. (G) Cycling performance of FeHCF||Zn hybrid batteries at 0.1 A g^−1^. Reproduced with permission [51]. Copyright 2022, Elsevier. (H) HRTEM image of the Mn@FeHCF. (I, J) Ex situ Raman patterns of the cycled electrodes (MnHCF and Mn@FeHCF) obtained at different cycles. (K) Cycling performance of Zn||MnHCF and Zn||Mn@FeHCF cells at 2 A g^−1^. Reproduced with permission [52]. Copyright 2023, American Chemical Society.

To address these challenges, employing Berlin green (FeHCF) instead of manganese hexacyanoferrate (MnHCF) has proven more effective due to its superior stability. Zhou and coworkers demonstrated that FeHCF, with its high crystallinity as the cathode material in ZPHIBs, maintained performance over 1000 cycles at 1 A g^−1^ [49]. Nevertheless, even in the absence of Jahn–Teller distortion, the FeHCF cathode experienced a notable capacity fade, dropping below 100 mAh g^−1^ within the first few cycles at 0.1 A g^−1^ (Figure 7A).

To enhance the stability of PBA cathodes, further strategies are necessary. Chen et al. reported a mixed electrolyte approach using propylene carbonate (PC) as a co‐solvent in the electrolyte of a MnHCF||Zn hybrid battery, serving dual purposes (Figure 7B) [50]. First, PC helps prevent the phase transition from MnHCF to ZnHCF, thereby preventing the two active sites of the cathode. This modification enabled a reversible capacity of 118 mAh g^−1^ and exhibited high cycling performance (Figure 7C). Ex situ X‐ray diffraction and ICP analyses at the 200th cycle demonstrated notable suppression of the phase transition (Figure 7D,E). Second, the inclusion of PC expanded the electrochemical stability window (the potential range over which an electrolyte can maintain stability), allowing the MnHCF||Zn hybrid battery to operate within a cut‐off voltage range of 1.1 V to 2.2 V. Similarly, FeHCF also showed promising cyclic stability in a mixed organic/water electrolyte. Zhou and coworkers incorporated non‐flammable triethyl phosphate (TEP) as a co‐solvent in the aqueous electrolyte to develop a FeHCF||Zn hybrid battery (Figure 7F) [51]. The hybrid electrolyte functioned similarly to the one previously described, and the FeHCF||Zn hybrid battery achieved a remarkable specific capacity of 142 mAh g^−1^ at a current density of 0.1A g^−1^ (Figure 7G). The above studies confirm that the addition of an organic solvent to the aqueous electrolyte greatly improves the cycle stability and inhibits adverse phase transitions. Most recently, Li et al. employed an organic/water hybrid electrolyte comprising 1 m K(CF_3_SO_3_) and 0.1 m Zn(CF_3_SO_3_)_2_ in a mixture of TEP and water at a volumetric ratio of 9:1 [52]. This electrolyte was used to design a core‐shell double‐atom‐redox PBA, which effectively suppressed Jahn–Teller distortion and minimized Mn dissolution (Figure 7H). By leveraging the lattice compatibility between MnHCF and FeHCF, a core‐shell binary hexacyanoferrate (Mn@FeHCF) was synthesized, exhibiting epitaxial growth and effectively impeding phase transitions (Figure 7I,J). Consequently, the assembled full cells showed a high capacity of 166 mAh g^−1^ and maintained a capacity retention of 83.9% over 4800 cycles at a current density of 2 A g^−1^ (Figure 7K).

## 6 Zinc‐Lithium and Zinc‐Ammonium Hybrid Ion Batteries

Prior research indicates that PBA cathodes are not optimal for aqueous lithium‐ion batteries primarily due to a mismatch between the size of solvated Li^+^ ions and the ion channels of PBA cathodes. This mismatch leads to incomplete intercalation and extraction of lithium ions during the charge and discharge cycles, leading to the degradation of the PBA cathode structure and potential severe dissolution of cathode materials in the aqueous electrolyte. However, recent studies have reported that ZLHIBs with PBA cathodes demonstrate promising cycling performance.

**FIGURE 8:**
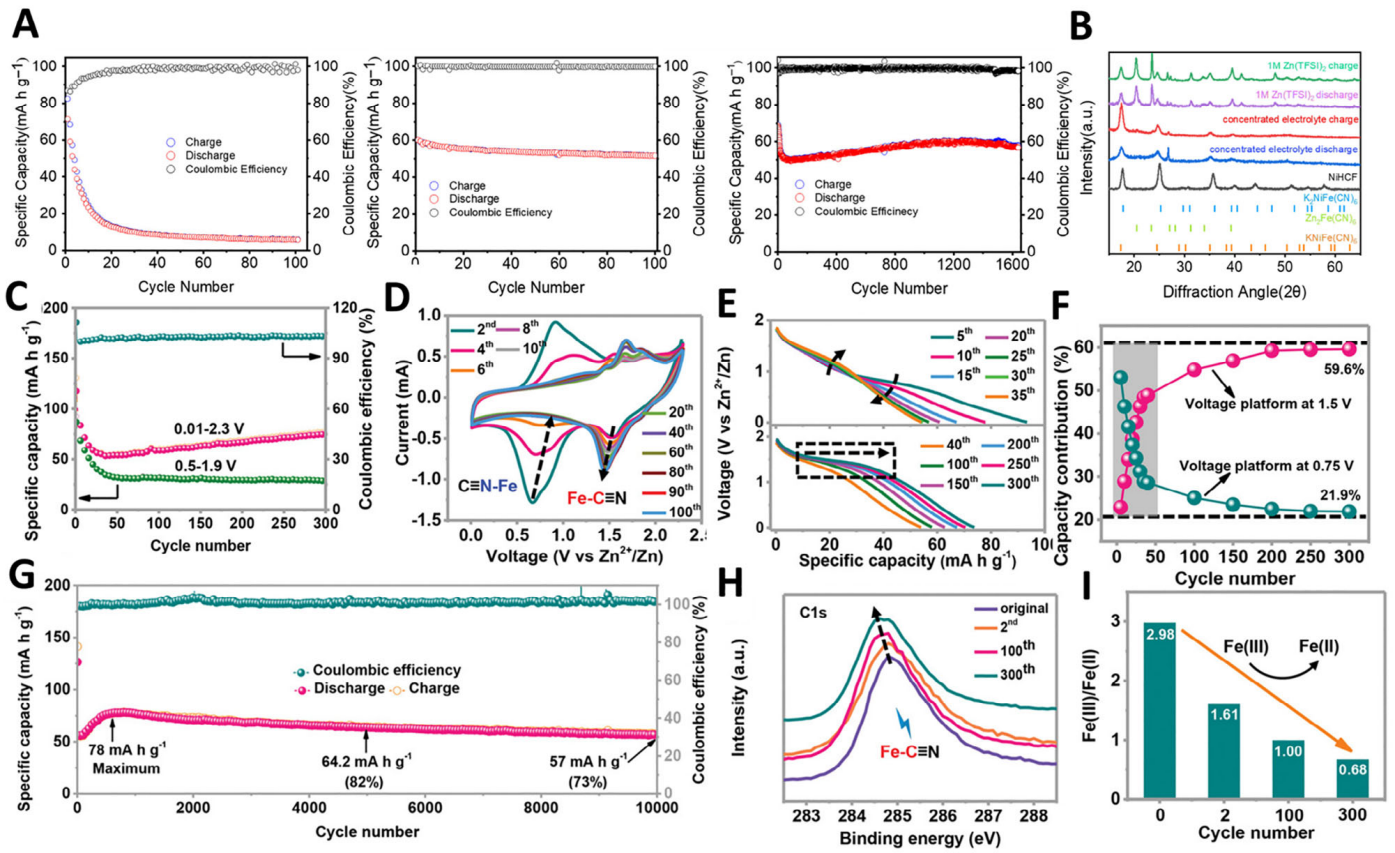
(A) Cycling performance of NiHCF‖Zn cells. (B) XRD patterns of NiHCF after 50 cycles in different electrolytes. Reproduced with permission [53]. Copyright 2020, Wiley. (C) Cycling performance of NiHCF‖Zn batteries operated in two different voltage windows. (D) In situ CV curves were recorded at 2 mV s^−1^. (E) GCD profiles and (F) the corresponding capacity contribution of two voltage platforms after various cycles at the current density of 1 A g^−1^. (G) Cycling performance of the FeHCF‖Zn cells. (H) Ex situ XPS of C 1s. (I) Evolution of Fe^III^/Fe^II^ ratio. Reproduced with permission [54]. Copyright 2019, Wiley.

Liang and coworkers examined the phase stability of NiHCF in a variety of electrolytes, including diluted electrolyte (1 m ZnSO_4_, or 1 m Zn(TFSI)_2_), organic electrolyte (0.5 m Zn(ClO_4_)_2_ in acetonitrile) and concentrated electrolyte (1 m Zn(TFSI)_2_ + 21 m LiTFSI). It is verified that water activity is critical factor affecting the stability of NiHCF (Figure 8A) [53]. High water activity results in severe nickel dissolution, structure collapse, rapid capacity decline, and formation of a new phase of Zn_2_Fe(CN)_6_ (Figure 8B). In contrast, in concentrated electrolytes, NiHCF maintained a reversible capacity of 60.2 mAh g^−1^ over 1600 cycles. Zhi and coworkers introduced a high‐voltage scanning activate method to maximize the utilization of the redox sites of the low‐spin Fe coordinated with the carbon atoms of the cyano group in ZLHIBs, using a high concentration mixed electrolyte (21 m LiTFSI + 1 m Zn(TFSI)_2_) [54]. As shown in Figure 8C–F, the low‐spin Fe was gradually activated at higher voltages, thus becoming the predominant contribution to capacity through two redox couples. Furthermore, ex situ X‐ray photoelectron spectroscopy (XPS) confirmed the continuous activation of low‐spin iron (Figure 8H,I). As a result, the FeHCF||Zn battery exhibited superior rate capability with a 73% capacity retention after 10,000 cycles at a current density of 3 A g^−1^ (Figure 8G).

**FIGURE 9:**
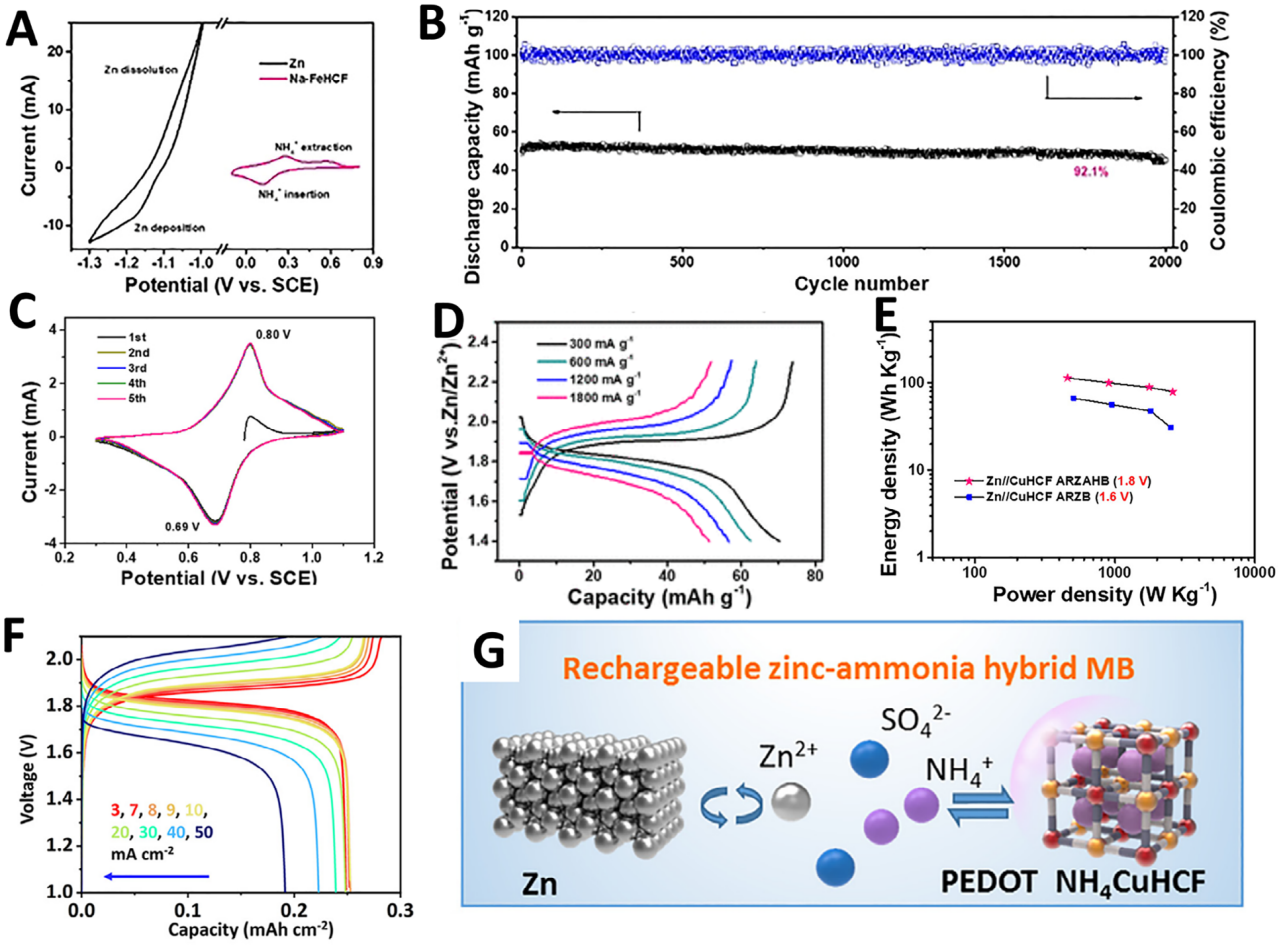
(A) CV curves of Zn and FeHCF electrodes in 1 M (NH_4_)_2_SO_4_ and 20 mM ZnSO_4_. (B) Cycling performance of the FeHCF||Zn cells. Reproduced with permission [55]. Copyright 2019, Wiley. (C) First five CV curves at 3 mV s^−1^ of CuHCF. (D) GCD profiles of CuHCF. (E) Ragone plots of CuHCF||Zn ZAHIBs with a comparison of CuHCF||Zn ZIBs. Reproduced with permission [57]. Copyright 2019, American Chemical Society. (F) Electrochemical performance of GCD curves of NH_4_CuHCF‐P||Zn MBs. (G) Schematic illustration of RZAHI‐MBs. Reproduced with permission [58]. Copyright 2023, Elsevier.

Aqueous rechargeable ammonium‐ion batteries are garnering great interest due to their high safety, environmental friendliness, and effectiveness. However, the lack of suitable anode materials has impeded their advancement. Recently, Huang and coworkers reported a new ZAHIBs with FeHCF as cathode, zinc as anode, and a mixture of 1 m (NH_4_)_2_SO_4_ and 0.02 m ZnSO_4_ as electrolyte [55]. These ZAHIBs possess an average voltage of 1.3 V and a long cycle life with 92.1% capacity retention after 2000 cycles at 2 A g^−1^ (Figure 9A,B). Despite their excellent rate and cycling performance, the low cell voltage of 1.3 V limits their energy density to below 100 Wh kg^−1^. To enhance the insertion potential of PBAs, one effective strategy is to modify the metal ion coordination with the Fe[CN]_6_ complex. Cui and coworkers discovered that incorporating copper ions significantly increases the redox potential of PBAs due to their strong inducing effect on the Fe^III^/Fe^II^ couple. This finding positions copper hexacyanoferrate (CuHCF) as a promising candidate with a higher redox potential [56]. Building on this, Huang and coworkers employed CuHCF as a cathode for ZAHIBs, achieving a high discharge voltage of 1.8 V and a satisfactory energy density of 114 Wh kg^−1^, with the batteries maintaining 76.5% capacity after 1000 cycles (Figure 9C–E) [57]. In a further advancement, Yang et al. developed a novel dual‐complexant electrodeposition technique to prepare a high‐mass‐loading NH_4_CuHCF cathode on porous nickel, which delivers a high discharge plateau of 1.8 V and a remarkable energy density of 0.44 mWh cm^−2^ (Figure 9F) [58]. To further improve the cycling stability and the electronic conductivity of the cathode, they also applied a coating of poly(3,4‐ethylenedioxythiophene) (PEDOT) to the NH_4_CuHCF (Figure 9G). As a result, the organic–inorganic hybrid cathode exhibits a robust rate capability of 100 mA cm^−2^ and maintains cycling stability over 1000 cycles in micro‐batteries (MBs). These developments underscore the potential of innovative cathode materials and electrode fabrication techniques in enhancing the performance of ammonium‐based hybrid ion batteries.

## 7 Summary and Outlook

**FIGURE 10:**
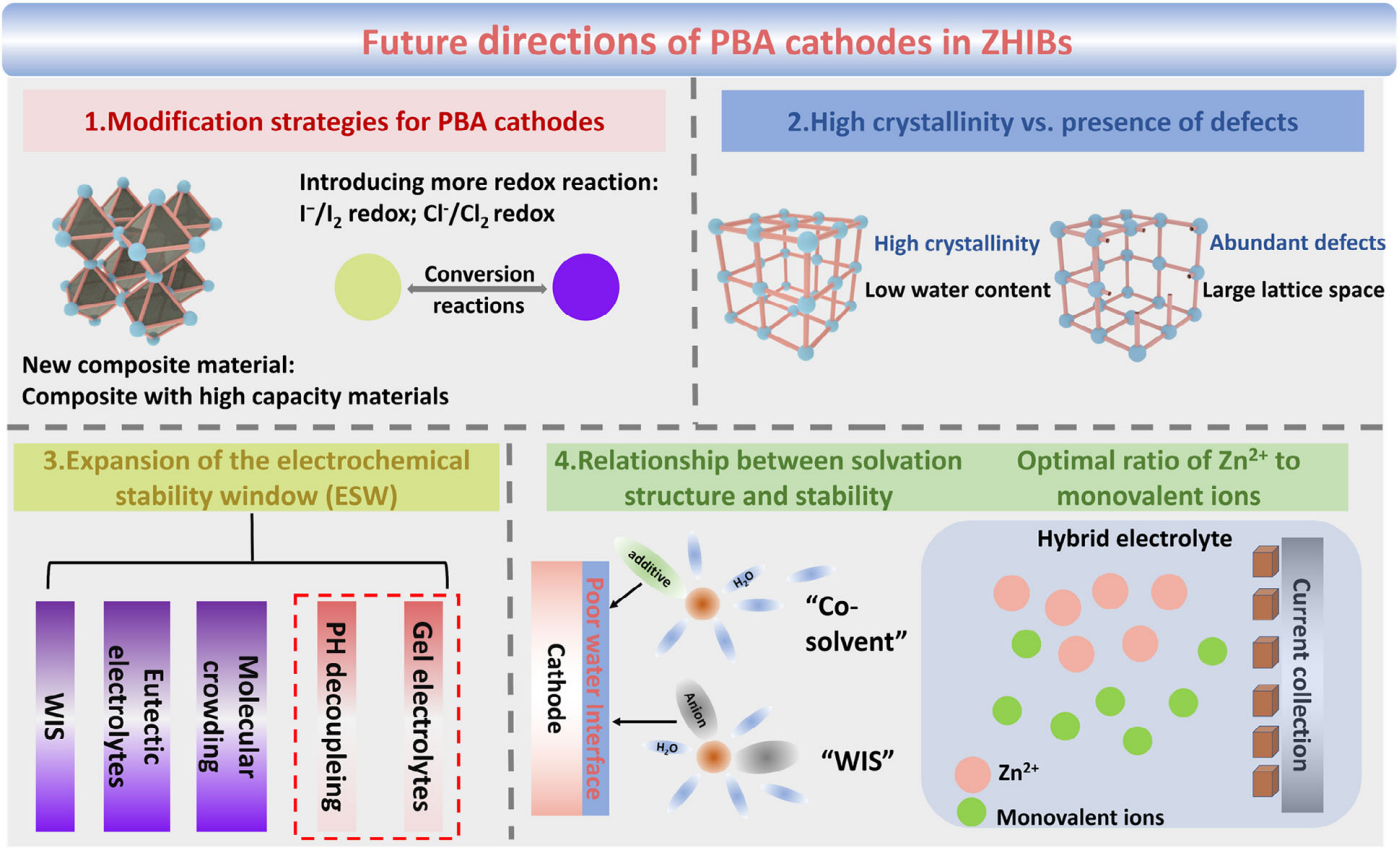
Future directions of PBA cathodes in ZHIBs.

Considering the escalating demand for electrochemical energy storage technologies, the development of advanced and safe batteries with earth‐abundant materials is of great importance. Capitalizing on the attributes of various monovalent elements as charge carriers and the merits of a zinc anode, zinc hybrid ion batteries (ZHIBs) have emerged as promising options for large‐scale energy storage applications. To date, PBA cathodes have attracted considerable attention due to their open framework with large channels, controllable structure, and cost effectiveness, positioning them as one of the most promising cathode materials for ZHIBs (Table 1). In this review, we summarize the challenges and recent advancement in PBA cathodes for various ZHIB configurations, including ZLHIBs, ZSHIBs, ZAHIBs, and ZPHIBs. Although considerable progress has been made in the application of PBA cathodes in ZHIBs in recent years, this field remains nascent with numerous unresolved issues and phenomena. We identify the following research directions and challenges, as illustrated in Figure 10.

**TABLE 1:** The type of cathode, composition and stability of the electrolyte and electrochemical performance of ZHIBs.

Cathode	Electrolyte	Electrochemical Window	Performance	Ref.
FeHCF	3 m Zn(OTf)_2_ + 3 m KOTf in water	0.6–2.0 V	57% after 1000 cycles (1 A g^−1^) 169.2 mAh g^−1^ (0.1 A g^−1^) ≈146.4 Wh kg^−1^ (0.1 A g^−1^)	[49]
ZnHCF	3 m KOTF + 2 m Zn(OTF)_2_ in water	1.0–2.2 V	89% after 2000 cycles (5 A g^−1^) 68.1 mAh g^−1^ (1 A g^−1^) ≈120.1 Wh kg^−1^ (1 A g^−1^)	[46]
ZnHCF	30 m KFSI +1 m Zn(OTF)_2_ in water	0.5–2 V	100% after 1000 cycles (0.4 A g^−1^) 68.9 mAh g^−1^ (0.2 A g^−1^) ≈124.02 Wh kg^−1^ (0.2 A g^−1^)	[61]
FeMnHCF	1 m KOTF + 1 m Zn(OTF)_2_ in 40% EG PAM@DM	0.6–2.3 V	14,500 cycles (2.66 A g^−1^) 160 mAh g^−1^ (0.3 A cm^−2^) ≈201.6 Wh kg^−1^ (1.87 A g^−1^)	[62]
MnHCF	30 m KFSI + 1 m Zn(OTF)_2_ in water	0.5–2.0 V	98% after 400 cycles (0.2 A g^−1^) 138 mAh g^−1^ (0.2 A g^−1^) ≈183.6 Wh kg^−1^ (0.2 A g^−1^)	[48]
MnHCF	0.5 m KOTF + 0.1 m Zn(OTF)_2_ in TEP/HFE (7/3 vol%)	0.7–2.2 V	91.6% after 100 cycles (20 mA g^−1^) 151 mAh g^−1^ (0.02 A g^−1^) ≈262.7 Wh kg^−1^ (20 mA g^−1^)	[35]
NiCoHCF	2 m KNO_3_ in water	1.0–2.2 V	93.1% after 50 cycles (0.1 A g^−1^) 52.6 mAh g^−1^ (1 A g^−1^) 96.81 Wh kg^−1^ (0.1 A g^−1^)	[63]
ZnHCF	0.5 m KOTF + 0.1 m Zn(OTF)_2_ in TEP	0.7–2.2 V	74% after 100,000 cycles (2 A g^−1^) 57 mAh g^−1^ (2 A g^−1^) ≈96.8 Wh kg^−1^ (2 A g^−1^)	[64]
FeMnHCF	4 m KOTF + 2 m Zn(OTF)_2_ in water	1.5–2.0 V	85.3% after 1000 cycles (1 A g^−1^) 62.4 mAh g^−1^ (0.5 A g^−1^) ≈118.4 Wh kg^−1^ (0.5 A g^−1^)	[65]
FeHCF	2 m ZnSO_4_ + 0.5 m Na_2_SO_4_ in SA‐PAM	0.8–1.4 V	73% after 10,000 cycles (20 C) 136.4 mAh g^−1^ (0.1 C) ≈143.2 Wh kg^−1^ (0.1 C)	[66]
NiHCF	1 m Na_2_SO_4_ + 0.02 m ZnSO_4_ in water	0.8–2.1 V	91% after 1000 cycles (0.5 A g^−1^) 73 mAh g^−1^ (0.1 A g^−1^) 99.1 Wh kg^−1^ (0.1 A g^−1^)	[67]
NiHCF	1 m ZnSO_4_ + 1 m Na_2_SO_4_ in water	0.7–1.85 V	75% after 200 cycles (0.2 A g^−1^) 73.9 mAh g^−1^ (0.1 A g^−1^) ≈92 Wh kg^−1^ (0.2 A g^−1^)	[40]
FeHCF	1 m Na_2_SO_4_ + 0.02 m ZnSO_4_ in water	0.7–2.1 V	75% after 800 cycles (0.25 A g^−1^) 141.6 mAh g^−1^ (0.1 A g^−1^) ≈132.5 Wh kg^−1^ (0.1 A g^−1^)	[68]
FeHCF	7 m NaOTF + 0.1 m Zn(OTF)_2_ in water/VC (98/2 vol%)	0.6–1.5 V	63% after 1700 cycles (1 C) 75 mAh g^−1^ (1 C) ≈74.5 Wh kg^−1^ (1 C)	[69]
FeHCF	21 m LiTFSI +1 m Zn(TFSI)_2_ in water	0.01–2.3 V	73% after 10,000 cycles (3 A g^−1^) 78 mAh g^−1^ (3 A g^−1^) ≈70 Wh kg^−1^ (1 A g^−1^)	[54]
CuHCF	0.5 m (NH_4_)_2_SO_4_ + 0.05 m Zn (OTF)_2_ in water	1.0–2.1 V	94% after 1000 cycles (20 mA cm^−1^) 0.59 mAh cm^−2^ (5 mA cm^−2^) 0.44 mWh cm^−2^	[58]
Na_3_V_2_(PO_4_)_3_	10 m NaClO_4_ + 0.17 m Zn(CH_3_COO)_2_ in water/VC (98/2 wt%)	0.8–1.7 V	84% after 1000 cycles (5 A g^−1^) 94 mAh g^−1^ (0.2 A g^−1^) ≈139.7 Wh kg^−1^ (0.2 A g^−1^)	[70]
V_2_CT*_X_*	21 m LiTFSI + 1 m Zn(CF_3_SO_3_)_2_ in water	0.1–2.0 V	/ 508 mAh g^−1^ (0.2 A g^−1^) 386.2 Wh kg^−1^ (0.2 A g^−1^)	[71]
Na_0.95_MnO_2_	0.5 m CH_3_COONa + 0.02 m Zn(CH_3_COO)_2_ in water	1.0–2.0 V	98% after 1000 cycles (4C) 60 mAh g^−1^ (2C) 78 Wh kg^−1^ (2C)	[72]

1. Modification strategies for PBA cathodes. PBAs exhibit a robust open framework structure that can accommodate a variety of metal and non‐metal ions. However, the structures of inherent frameworks, typically comprising transition metals linked by coordination bonds to cyanide groups, are challenging to alter. As a result, current design approaches for PBA cathodes are somewhat limited, often focusing on doping at the transition metal site to create high entropy modifications and similar adjustments. The difficulty in altering the composition also restricts the specific capacity of the PBA cathodes, which seldom exceeds 200 mAh g^−1^. Therefore, it is imperative to develop novel modification method for PBA cathodes, such as introducing additional redox pairs or integrating them with high‐capacity cathode materials to boost their capacity.
2. High crystallinity versus presence of defects. A major challenge for PBA cathodes, particularly in the context of sodium‐ion batteries, is their inherent moisture content (approximately 10 wt%), which significantly impacts their high‐temperature storage performance and cycle life. While most strategies aimed at improving crystallinity involve the use of chelating agents to slow down the crystallization process, the impact of ligands and water during synthesis is often neglected. Approaches such as in situ vacancy repair, ball milling, and solid‐state synthesis can yield highly crystalline PBAs. However, in aqueous systems, lattice defects, which may arise from increased coordination of PBA with water molecules, could facilitate ion intercalation and extraction by enlarging the lattice spacing. Therefore, in future research, it is crucial to develop PBA cathodes with minimal defects for ZHIBs. [59]
3. Expansion of the electrochemical stability window (ESW). PBA cathodes operate at high voltages where a narrow ESW can lead to electrolyte decomposition and oxygen evolution, causing battery swelling and low coulombic efficiency. Various methods to widen the water voltage window have been developed, such as WIS electrolytes, introduction of organic diluents, eutectic electrolytes, decoupled batteries, and gel electrolytes [60]. Among them, the high cost of “WIS” is often disregarded. Moreover, eutectic electrolytes and organic diluents require the introduction of a large number of organic components into aqueous electrolytes, necessitating a careful balance between ESW, cost, and ionic conductivity. Given the stability of PBAs in acidic environments, utilizing decoupled systems to broaden the voltage window might be a feasible approach and worth more thorough investigation in the future. In addition, the development of gel electrolytes with reduced water activity is another promising way to broaden the ESW.
4. Relationship between solvation structure and stability. Many studies have shown that PBAs in “WIS” systems or mixed organic/water solvent electrolytes endow assembled zinc‐based batteries with superior cycle stability. The common feature of these systems is the altered solvation structure of the electrolyte, which reduces the water content in the inner layer. However, in‐depth studies on the mechanism of these electrolyte systems are lacking, and the exact mechanism underlying their stability improvements remains undefined. Besides, the ratio of Zn^2+^ to monovalent ions needs to be further investigated. While it is commonly believed that monovalent ions are preferable for intercalation and extraction processes, it represents a meaningful research direction for adjusting the optimal ratio of Zn^2+^ to monovalent ions in the electrolyte configuration, to simultaneously achieve efficient utilization of the cathode and stable zinc deposition and dissolution on the zinc anode side.

## Conflicts of Interest

The authors declare no conflicts of interest.
